# Asynchronous movement of sarcomeres in myocardium under living conditions: role of titin

**DOI:** 10.3389/fphys.2024.1426545

**Published:** 2024-08-02

**Authors:** Fuyu Kobirumaki-Shimozawa, Kotaro Oyama, Tomohiro Nakanishi, Shin’ichi Ishiwata, Norio Fukuda

**Affiliations:** ^1^ Department of Cell Physiology, The Jikei University School of Medicine, Minato-ku, Tokyo, Japan; ^2^ Takasaki Institute for Advanced Quantum Science, National Institutes for Quantum Science and Technology, Takasaki-shi, Gunma, Japan; ^3^ Department of Anesthesiology, The Jikei University School of Medicine, Minato-ku, Tokyo, Japan; ^4^ Department of Physics, Faculty of Science and Engineering, Waseda University, Shinjuku-ku, Tokyo, Japan

**Keywords:** cardiac muscle, connectin, contractility, myofibril, passive force

Myocardial performance is highly dependent on sarcomere length (SL), namely, SL-dependent activation, a basis of the Frank-Starling law of the heart (Kobirumaki-Shimozawa et al., 2014; Kurihara and Fukuda, 2024). Indeed, a change in SL by only ∼100 nm (∼5% of SL) markedly increases active force in myocardial preparations, especially under physiologic partial activation conditions (see Kobirumaki-Shimozawa et al., 2014 and references therein).

Physiologists have long considered that sarcomeres in striated muscle, either cardiac or skeletal, shorten and lengthen in synchrony during excitation-contraction coupling (e.g., Kurihara and Fukuda, 2024). However, advances in recent technologies enabling high-speed high-resolution imaging of sarcomere dynamics pose an amendment to this traditional view, providing evidence that the lengths of individual sarcomeres vary at rest and do not change in a synchronous fashion throughout the contraction cycle even along the same myofibrils. For example, by using isolated intact guinea-pig ventricular myocytes, Lookin et al. (2022) reported that **1)** the variation of SL (median deviation divided by the average SL) is ∼4%, and **2)** the variability in end-systolic SL is ∼1.3-fold greater as compared to end-diastolic SL. These findings are in good agreement with those obtained in our previous study in ventricular myocytes in the beating heart of healthy mice, where the variation values were 4.6% and 5.6% during diastole and systole, respectively (Kobirumaki-Shimozawa et al., 2016). SL inhomogeneity can be seen in skeletal muscle as well under living conditions; namely, in the tibialis anterior muscle of mice, the variation of resting SL is ∼4%, and it increases to ∼5% and ∼8% in short (180 ankle flexion) and long muscles (90° ankle flexion), respectively, during contraction (Moo et al., 2017). It can therefore be concluded that in either cardiac or skeletal muscle, there exists a certain magnitude of SL inhomogeneity at rest under living conditions, and accordingly, **1)** individual sarcomeres move in a rather asynchronous fashion during contraction, and **2)** their average movement, caused as a result of inter-sarcomere interactions along myofibrils, underlies cellular and muscular dynamics.

What is the mechanism of asynchronous behaviors of sarcomeres? It is important to clarify this issue because asynchronous SL movement in myocardium is expected to reduce the heart’s contractility. One possibility is that [Ca^2+^]_i_ changes vary locally in myocytes, in that the amount of Ca^2+^ released from the sarcoplasmic reticulum may differ along myofibrils. To test this possibility, we simultaneously measured the changes in local [Ca^2+^]_i_ and SL in rat neonatal cardiomyocytes (Tsukamoto et al., 2016). We found that while local [Ca^2+^]_i_ changes occur in a synchronous fashion along myofibrils, the movements of individual sarcomeres vary markedly. This finding indicates that the asynchronous movements of sarcomeres are intrinsic to the contractile system *per se*, and not related to the variation of intracellular Ca^2+^ dynamics in myocytes.

Other investigators have likewise reported that SL inhomogeneity can impact intra-myofibrillar sarcomeric interactions, thereby altering the dynamic performance of striated muscles. First, a single half-sarcomere-based mathematical model successfully simulated half-sarcomere length inhomogeneity and, concomitantly, active force responses in rabbit fast skeletal muscle fibers under conditions where length changes occur (Campbell, 2009). By using the same model, Campbell et al. (2011) showed that half-sarcomere inhomogeneity underlies “residual force enhancement” that appears when rabbit fast skeletal muscle fibers are stretched during contraction, suggesting that the phenomenon reflects mechanical interactions between half-sarcomeres with inhomogeneous lengths [later, experimentally demonstrated by Rassier and Pavlov (2012)]. Second, Stehle et al. (2002) reported that, upon a rapid decline in the Ca^2+^ concentration, relaxation occurs at the single sarcomere level and it propagates from sarcomere to sarcomere along isolated myofibrils from guinea pig left ventricles. This single sarcomere-based, stretch-dependent propagation of relaxation was predicted earlier by Brutsaert et al. (1984), and it is similar to that observed during sarcomeric auto-oscillations (i.e., SPOCs: see Kagemoto et al., 2018; Harris, 2021 and references therein) occurring at partial Ca^2+^ activation in permeabilized skeletal and cardiac muscles. Tangney et al. (2014) provided consistent experimental evidence that physiological stretches during the falling phase of Ca^2+^ transient dissociates ∼50% of attached cross-bridges (hence deactivation) in intact mouse ventricular muscle.

We developed a quantification method for the magnitude of synchrony of sarcomere movement by introducing the parameter “Contribution Index (CI),” defined based on correlation coefficient matrices [from −1 (complete negative correlation) to 1 (complete positive correlation); for details see Kobirumaki-Shimozawa et al., 2021] (Figure 1A). In that previous study, imaging was performed at a spatial resolution of 10 nm at 100 frames per second (fps) for ventricular myocytes in the beating mouse heart *in vivo*. Under the physiological condition with ΔLVP (difference in left ventricular pressure in diastole and systole) of 96.4 mmHg, CI between adjacent sarcomeres varied markedly from ∼−0.5 to ∼0.5, with the average of −0.06 ± 0.28 (average ± SD) (Figure 1B). This negative value shows that adjacent sarcomeres tend to move in different directions. Individual sarcomere movements were, however, in positive correlation with the average motion of all sarcomeres along a myofibril; namely, CI between an individual sarcomere and the average of all sarcomeres was 0.29 ± 0.20 at ΔLVP 96.4 mmHg, i.e., a positive value (CI increased to 0.48 ± 0.24 at ΔLVP 135.7 mmHg) (Figures 1C, D). Moreover, 72% of sarcomeres showed CI > 0.2 at ΔLVP 96.4 mmHg, i.e., a positive contribution to the overall myofibrillar dynamics (the percentage increased to 88% at ΔLVP 135.7 mmHg) (Figures 1E, F; Kobirumaki-Shimozawa et al., 2021). These lines of evidence demonstrate that while the movements between adjacent sarcomeres are rather asynchronous, ∼70% of sarcomeres move in a synchronous fashion during the cardiac cycle, based on our definition, along myofibrils in the ventricle of the normal healthy mouse heart. Moreover, CI for each sarcomere varied between cardiac cycles, indicating that sarcomeres alternately contribute to myofibrillar dynamics (Kobirumaki-Shimozawa et al., 2021). Under living conditions, therefore, the magnitude of contribution of a sarcomere is determined by dynamic force balance along a myofibril.

**FIGURE 1:**
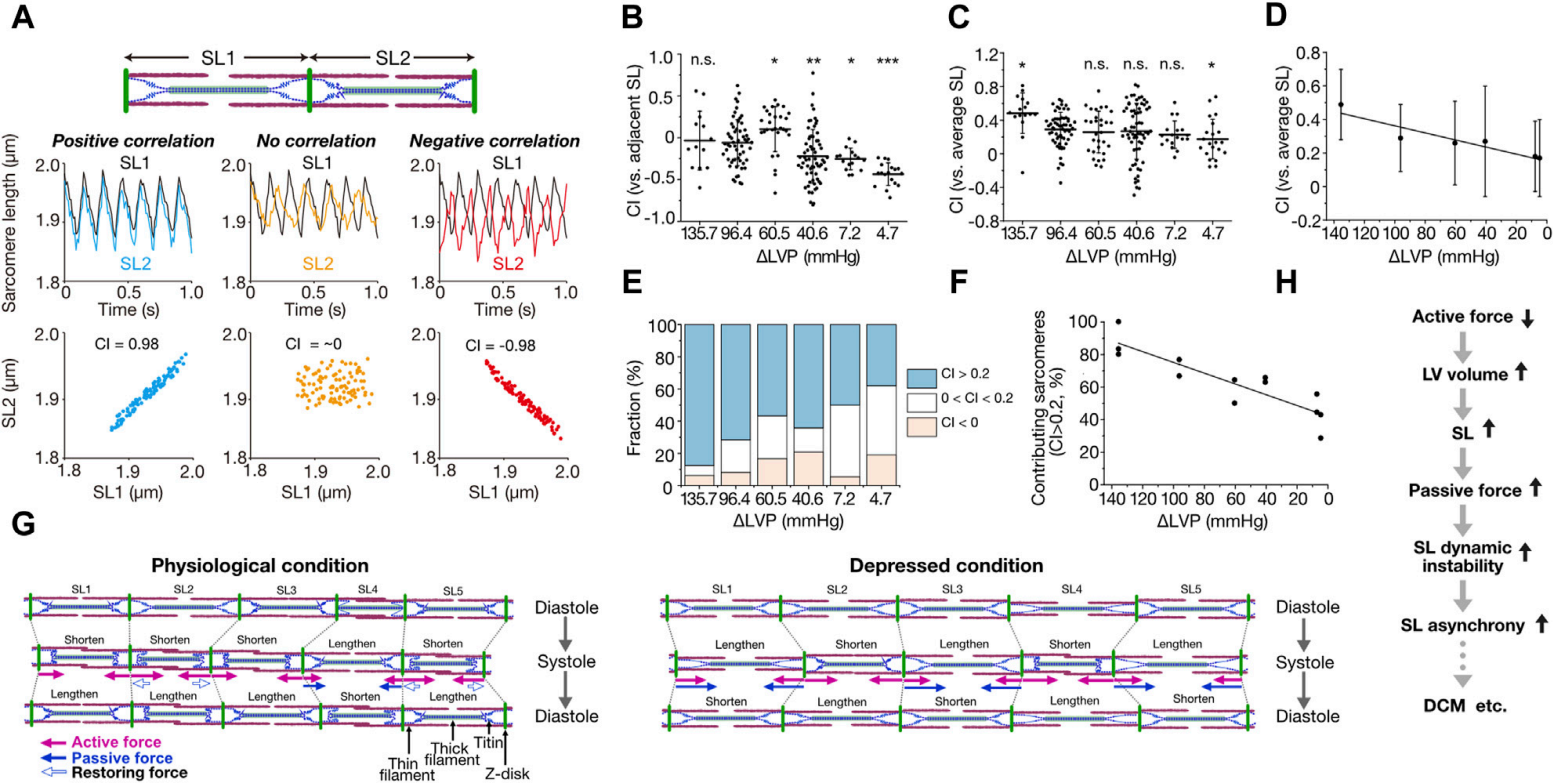
Asynchronous movement of sarcomeres in myocardium under living conditions. **(A)** Schematic showing the correlation analysis of the movements of two adjacent sarcomeres. SL1 and SL2 indicate the sarcomeres on left and right, respectively. When both sarcomeres contract and relax in a synchronized fashion, contribution index (CI) defined as the correlation coefficient between SL1 and SL2 becomes closer to 1 (0.98 in this theorical case). When both sarcomeres move independently, CI becomes closer to 0. Virtual SL waveforms of 5–7 Hz were plotted at a rate of 100 fps. **(B)** Graph showing CI between adjacent sarcomeres along myofibrils at various ΔLVP. Horizontal bars, average; vertical bars, SD. Not significant (n.s.), *P* > 0.05; *P < 0.05; **P < 0.01; and ***P < 0.001 compared with 96.4 mmHg. **(C)** Graph showing CI between each sarcomere and the average of all sarcomeres along myofibrils at various ΔLVP. Horizontal bars, average; vertical bars, SD. Not significant (n.s.), *P* > 0.05; *P < 0.05 compared with 96.4 mmHg. **(D)** Same as in **(C)** with a linear regression line. A significant (*P* < 0.05) linear relationship exists. **(E)** Graph showing the fraction of sarcomeres contributing to myofibrillar dynamics at various ΔLVP. CI between each sarcomere and the average of all sarcomeres along myofibrils was categorized as follows: blue (CI > 0.2), white (0 < CI < 0.2) and red (CI < 0). **(F)** Relationship of the fraction of contributing sarcomeres (CI > 0.2) vs. ΔLVP. A significant (*P* < 0.05) linear relationship exists. See Kobirumaki-Shimozawa et al., 2021 for details on the experimental data in **(B–F)**. **(G)** Schematic illustrating the mechanism of intramyofibril interactions. Left: Physiological condition. SL is inhomogeneous during diastole. Upon systole, SL2 and SL5 exhibit greater shortening than SL1 and SL3 because of their longer SL via predominantly length-dependent activation, and pull the neighboring SL4. Then, lengthened SL4 produces inward-oriented passive force. In subsequent diastole, shortened sarcomeres (SL1, SL2, SL3 and SL5) lengthen due, at least in part, to outward-oriented, titin-based restoring force (especially SL2 and SL5), but SL4 shortens because of inward-oriented passive force, consequently causing asynchrony. Right: Depressed condition in which ventricular volume is increased due to impaired systolic function. In diastole, sarcomeres with longer lengths (SL2 and SL4) exhibit greater passive force and greater SL-dependent activation, and shorten upon systole at the expense of shorter sarcomeres, which are lengthened accordingly (SL1, SL3 and SL5). Thus, titin-based passive force (that can surpass active force) markedly increases in SL1, SL3 and SL5, causing them to abruptly shorten upon subsequent diastole, stretching their adjacent neighbors (SL2 and SL4). Consequently, sarcomeres move in marked asynchrony. In these illustrations, arrows indicate the forces operating in systole: namely, pink closed arrows, actomyosin-based active force; blue closed and open arrows, titin-based passive and restoring forces, respectively. **(H)** Flowchart showing the postulated role of SL asynchrony under diseased conditions. Reduction of myocardial contractile force results in an increase in ventricular volume, elongating SL and thereby increasing titin-based passive force. Enhanced instability in the force balance between sarcomeres causes SL asynchrony and exacerbates cardiac pump function in coordination with impaired contractile force. **(B–G)**, modified based on Kobirumaki-Shimozawa et al. (2021) with permission.

The average CI is relatively low in the range of ∼0.3–0.5 under normal or slightly high contractile conditions (Figures 1C, D). Provided that diastolic SL is usually ∼2.0 μm in the ventricle of the beating heart in mice (Kobirumaki-Shimozawa et al., 2016; Kobirumaki-Shimozawa et al., 2021), we consider that titin (connectin) plays a pivotal role in the regulation of individual sarcomeric movements. This is because stiff N2B titin (but not more compliant N2BA titin) is dominantly expressed in the mouse ventricle (see Kobirumaki-Shimozawa et al., 2014 and references therein), and, reportedly, a change in SL by only ∼100 nm can substantially affect passive as well as active properties (the latter operating via a change in interfilament lattice spacing; see Kobirumaki-Shimozawa et al., 2014 and references therein). Namely, as long as there is a certain magnitude of SL inhomogeneity along myofibrils in diastole, sarcomeres of relatively long lengths will pull adjacent shorter neighbors (producing less active force) in subsequent systole (Figure 1G). This is because SL-dependent activation is preserved at the single sarcomere level in the beating heart *in vivo* (Kobirumaki-Shimozawa et al., 2021). Then, sarcomeres switch between roles in subsequent diastole, namely, long sarcomeres become shorter than their neighbors, resulting in alternant contribution to myofibrillar dynamics (as observed in Kobirumaki-Shimozawa et al., 2021). In sarcomeres that are shorter than the slack length (i.e., ∼1.90 μm in N2B titin-expressing mouse cardiomyocytes; e.g., Fukuda et al., 2008), outward-oriented restoring force is likely to develop in diastole, elongating these sarcomeres to the slack SL range (Figure 1G). Therefore, under conditions where diastolic SL varies along a myofibril, such as in the beating heart *in vivo*, sarcomeric movements cannot be in complete synchrony but become rather asynchronous with CI ∼0.3–0.5.

If the imbalance of titin’s force between sarcomeres underlies their asynchronous movements, the magnitude of asynchrony should be increased under depressed conditions where SL is elongated to or beyond the (near) upper limit (∼2.30 µm for N2B titin) and titin-based passive force can surpass active force. Indeed, titin-based passive force increases in an exponential manner beyond SL ∼2.20 µm in N2B titin-expressing myocardium (see Kobirumaki-Shimozawa et al., 2014 and references therein). We mimicked this condition in living mice by increasing the percentage of isoflurane (from 2% to 5%), i.e., under deep anesthesia (Kobirumaki-Shimozawa et al., 2021). When 5% isoflurane was administered in mice, diastolic SL was elongated to >2.30 µm. As expected, CI between adjacent neighbors decreased to −0.25 ± 0.13 and −0.43 ± 0.13, and the average CI values were 0.23 ± 0.16 and 0.17 ± 0.24 at ΔLVP 7.2 and 4.7 mmHg, respectively (Figures 1B, C). Under this depressed condition, we consider that titin-based passive force *per se* operates to stretch adjacent sarcomeres, and causes marked asynchronous movements of sarcomeres (Figure 1D).

Recently, Li et al. (2023) stretched isolated intact rat and mouse ventricular myocytes in a step-by-step manner. They found that long sarcomeres pull weaker-active force producing shorter neighbors by taking advantage of greater active force (not titin-based passive force *per se*) during contraction upon stretch, recruiting them to stronger-active force producing long sarcomeres (and accordingly, increasing total isometric active force produced by myocytes). This observation appears to contradict the findings of our previous study under depressed conditions in the beating heart (Kobirumaki-Shimozawa et al., 2021), and therefore, a comment needs to be addressed; namely, in the work by Li et al. (2023), myocyte length was isometrically varied under the normal activating condition in the presence of 1.8 mM [Ca^2+^]_o_, and therefore, titin-based passive force is unlikely to surpass active force. We consider that the phenomenon discovered by Li et al. (2023) is, in principle, similar to what we observed at normal or slightly high ΔLVP, in that long sarcomeres stretch shorter adjacent neighbors in systole by taking advantage of SL-dependent activation (Kobirumaki-Shimozawa et al., 2021); accordingly, total cardiomyocyte active force increases as a result of an increase in the fraction of long sarcomeres.

What is the perspective of asynchronous sarcomere movement in the beating heart, towards understanding the mechanism of impaired systolic function, such as in dilated cardiomyopathy (DCM)? Currently, no consensus has been achieved regarding whether or not impairment of sarcomeric function underlies depressed systolic dysfunction in DCM (see Kagemoto et al., 2018 and references therein). Based on the discussions above, we hereby propose that sarcomere asynchrony plays a role, at least in part, in the impaired systolic function under diseased conditions including DCM (Figure 1H). Namely, impaired systolic function (i.e., active force reduction) results in an increase in the ventricular volume due to blood congestion. Accordingly, SL is elongated (Kobirumaki-Shimozawa et al., 2021), and a high level of titin-based passive force will be generated, surpassing active force. Under this condition, sarcomeres are under dynamic instability along myofibrils due to enhanced tug-of-war between adjacent neighbors, causing marked asynchrony that can progressively exacerbate myocardial contractility (Figure 1H). It has been reported that titin isoform switches occur in DCM, namely, the N2BA titin/N2B titin expression ratio increases with the progression of DCM (e.g., Fukuda et al., 2008 and references therein). This isoform switching may be a compensatory mechanism to weaken dynamic instability in the force balance between connected sarcomeres.

In summary, recent nano-imaging studies have revealed that the length of each sarcomere does not change in a synchronous fashion throughout excitation-contraction coupling in myocytes in the beating heart *in vivo* (Kobirumaki-Shimozawa et al., 2016; Kobirumaki-Shimozawa et al., 2021), as well as in cultured (Shintani et al., 2014; Tsukamoto et al., 2016) or isolated cardiomyocytes (Li et al., 2023). Increasing levels of titin-based passive force under depressed conditions enhances dynamic instability between sarcomeres along myofibrils (Kobirumaki-Shimozawa et al., 2021), due presumably to blood congestion in the heart, which is likely to cause marked asynchrony of sarcomere movements and hence progressive exacerbation of myocardial contractility. Previous studies show that the slack SL is similar between myocytes with and without DCM (e.g., Inoue et al., 2013; Beqqali et al., 2016); however, no systematic studies have been conducted to accurately analyze the movements of individual sarcomeres in diseased myocardium, including DCM, under living conditions *in vivo*. Therefore, future studies are warranted to directly investigate the link between sarcomere asynchrony and cardiac pump function in various heart disease mouse models, especially DCM and congestive heart failure.

## Abbreviations

CI, contribution index; DCM, dilated cardiomyopathy; LVP, left ventricular pressure; SL, sarcomere length.
